# Effects of romosozumab and teriparatide on hip bone using 3D-SHAPER in postmenopausal women with osteoporosis

**DOI:** 10.1093/jbmrpl/ziaf151

**Published:** 2025-09-17

**Authors:** E Michael Lewiecki, Donald Betah, Ludovic Humbert, Cesar Libanati, Mary Oates, Yifei Shi, Renaud Winzenrieth, Serge Ferrari, Fumitoshi Omura

**Affiliations:** New Mexico Clinical Research & Osteoporosis Center, 300 Oak St NE, Albuquerque, NM 87106, United States; Amgen Inc., One Amgen Center Drive, Thousand Oaks, CA 91320, United States; 3D-SHAPER Medical, Rambla de Catalunya, 53, 4-H, Eixample, 08007 Barcelona, Spain; UCB, Allée de la Recherche, 60, Brussels B-1070, Belgium; Amgen Inc., One Amgen Center Drive, Thousand Oaks, CA 91320, United States; Amgen Inc., One Amgen Center Drive, Thousand Oaks, CA 91320, United States; 3D-SHAPER Medical, Rambla de Catalunya, 53, 4-H, Eixample, 08007 Barcelona, Spain; Division of Bone Diseases, University Hospital of Geneva, Geneva 1211, Switzerland; Koenji Orthopedics Clinic, 4-29-2, Koenji minami, Suganami-ku, Tokyo, 166-003, Japan

**Keywords:** cortical bone, drug treatment, fracture, osteoporosis, trabecular bone

## Abstract

DXA-based 3D-modeling (3D-DXA) of the hip was used to assess estimated changes in bone integral, cortical, and trabecular parameters and analyze 3D anatomical distribution of changes in bone parameters over time in women who transitioned to romosozumab or teriparatide following treatment with oral bisphosphonates in STRUCTURE and in treatment-naïve women in a romosozumab phase 2 dose-ranging study (Phase 2 study). Data from women who had hip DXA scans at baseline and months 6 and 12 in STRUCTURE or baseline and months 3, 6, and 12 in the Phase 2 study and provided consent for future research were analyzed. 3D-SHAPER software was applied to generate patient-specific 3D-models from hip DXA scans. Percentage changes from baseline in areal BMD (aBMD), integral volumetric BMD (vBMD), cortical thickness, cortical vBMD, cortical surface BMD (sBMD), and trabecular vBMD were evaluated. Data of 308 women from STRUCTURE (romosozumab, 160; teriparatide, 148) with 3D-DXA assessments at baseline and months 6 and 12 were analyzed. Greater increases in aBMD, integral vBMD, cortical thickness, cortical vBMD, cortical sBMD, and trabecular vBMD were seen by month 6 following treatment with romosozumab vs teriparatide (*p* < .001); additional gains were observed through month 12 (*p* < .001). Conversely, teriparatide treatment led to loss in cortical thickness, cortical vBMD, and cortical sBMD. Data of 70 women (romosozumab, 22; teriparatide, 23; placebo 25) from the Phase 2 study with 3D-DXA assessments at baseline and months 3, 6, and 12 were analyzed. Greater increases in cortical thickness, cortical vBMD, cortical sBMD, and trabecular vBMD were observed with romosozumab vs teriparatide or placebo. In summary, results from 3D-DXA analysis of DXA images from STRUCTURE and the Phase 2 study support established evidence that romosozumab improves hip cortical bone density and structure in treatment-naïve patients or patients previously treated with bisphosphonates, thereby contributing to the rapid antifracture efficacy of romosozumab.

## Introduction

Romosozumab is a bone-forming agent with the dual effect of increasing bone formation and decreasing bone resorption^1^^,^^2^ and is approved worldwide for the treatment of osteoporosis. Monthly subcutaneous romosozumab 210 mg for 12 mo resulted in larger gains in areal BMD (aBMD) at the LS and TH compared with placebo, alendronate, or teriparatide, as determined by two-dimensional (2D) DXA.^1^^,^^3^ These gains in aBMD with romosozumab reduced the risk of fractures, compared with those with placebo in the FRAME study^4^ and alendronate in the ARCH study.^5^ The efficacy and safety of romosozumab upon transitioning from bisphosphonate therapy was evaluated in the STRUCTURE study.^6^

STRUCTURE was a phase 3 clinical trial evaluating the safety and efficacy of romosozumab vs teriparatide in postmenopausal women with osteoporosis who had received oral bisphosphonate therapy for ≥3 yr and alendronate for ≥1 yr prior to screening.^6^ Significantly greater gains in hip and spine aBMD and estimated hip strength were observed with romosozumab vs teriparatide over 12 mo in patients at high risk for fracture transitioning from bisphosphonates.^6^ The use of QCT to determine the effects of treatments on BMD changes in the cortical vs trabecular compartments also showed significant gains in integral and cortical volumetric BMD (vBMD) at the hip with romosozumab but not with teriparatide over 12 mo. Gains in trabecular vBMD were larger with romosozumab but did not reach statistical significance between the treatment groups.^6^

While QCT is very useful in differentiating between the effects of therapies on bone compartments, it is not widely available in clinical practice and involves greater exposure to ionizing radiation than DXA. DXA-based three-dimensional (3D) modeling using the 3D-SHAPER software (3D-DXA) can potentially overcome these limitations of QCT. 3D-DXA uses a validated statistical 3D shape and density model of the proximal femur built from a database of QCT scans from Caucasian men and women to generate a patient-specific 3D model using a standard hip DXA scan.^7–9^ 3D-DXA also provides a way to map and display the distribution of bone changes on cortical vs trabecular compartments for studies with no QCT measurements and to generate outputs for clinicians to visualize and monitor the effects of osteoporosis treatment.^10–12^

Validation studies for 3D-DXA have been performed, with high correlation coefficients (*R*) reported between 3D-DXA and QCT measurements for vBMD of the cortical bone (*R* = 0.93) and trabecular bone (*R* = 0.86).^7^ In addition, 3D-DXA measurements were found to be associated with the incidence of hip fracture.^13^^,^^14^ Further, 3D-DXA has been used to demonstrate the effects of abaloparatide on hip cortical vBMD and bone strength,^10^ the effects of abaloparatide and teriparatide on hip cortical vBMD,^12^ and the effects of sequential therapy with abaloparatide followed by alendronate on the proximal femur in postmenopausal women with osteoporosis.^9^^,^^11^

Results from a post hoc analysis using 3D-DXA images from the ARCH and FRAME studies to assess estimated changes in hip integral, cortical, and trabecular bone parameters have been previously published.^9^ Results from the ARCH study showed greater gains in bone parameters with romosozumab vs alendronate treatment for month 12, with the gains sustained after follow-up treatment with open-label alendronate for another 12 mo. Results from the FRAME study showed greater gains in bone parameters after treatment with romosozumab vs placebo for 12 mo, with the gains sustained after follow-up treatment with denosumab for another 12 mo. Another study had reported differential effects of romosozumab and teriparatide on cortical bone.^6^ Here, we report the results from a post hoc analysis using 3D-DXA to assess estimated changes in hip integral, cortical, and trabecular bone parameters and analyze the anatomical distribution of 3D changes in bone parameters over time in women previously treated with bisphosphonate before being treated with romosozumab or teriparatide in STRUCTURE^6^ and in treatment-naïve women treated with romosozumab compared with placebo or teriparatide in a romosozumab dose-ranging phase 2 study (Phase 2 study).^1^ As such, this analysis adds new insights into the effects of romosozumab on the cortical and trabecular compartments in patients transitioning from bisphosphonates (STRUCTURE),^6^ compared with those with no prior treatment (Phase 2)^1^ as well as the previously published data from ARCH and FRAME.^9^

## Materials and methods

### Study designs and patients

The study designs for STRUCTURE^6^ (NCT01796301) and the romosozumab Phase 2 dose-ranging study (Phase 2 study) (NCT00896532)^1^ have been previously reported and are illustrated in Figure S1. STRUCTURE and the phase 2 dose-ranging study were conducted in accordance with the International Council for Harmonisation Good Clinical Practice guidelines and the principles of the Declaration of Helsinki. The protocol and amendments were approved by the institutional review board at each participating site and regulatory authorities of participating countries. All patients provided written informed consent.

For each of the two trials, data from a subset of selected women per treatment group who had completed the 12-mo study period, provided consent for future research, and had TH DXA scans at baseline and at the time of interest (months 6 and 12 in STRUCTURE; months 3, 6, and 12 in the Phase 2 study) were included in the current post hoc analysis.

STRUCTURE^6^ enrolled 436 postmenopausal women with osteoporosis aged 55-90 yr who had received oral bisphosphonate therapy for ≥3 yr and oral weekly alendronate (70 mg or equivalent) for ≥1 yr prior to screening. Eligible women had T-score ≤−2.5 at the TH, FN, or LS; a history of nonvertebral fracture after age 50 yr or vertebral fracture at any time; and ≥1 hip and ≥2 vertebrae in the L1-L4 region evaluable by DXA.^6^ Women were randomly assigned to receive open-label monthly subcutaneous romosozumab 210 mg (218 women) or daily subcutaneous teriparatide 20 μg (218 women) for 12 mo (Figure S1A). The primary endpoint was percentage change from baseline in aBMD by DXA at the TH through month 12, and key secondary endpoints included percentage change from baseline through month 12 in cortical and integral vBMD by QCT.^6^ aBMD and vBMD were assessed at baseline and months 6 and 12. Results for the primary and secondary endpoints have been previously published.^6^ In the current analysis, 3D-DXA was performed on data from women enrolled in STRUCTURE who had completed the 12-mo study period, had provided consent for future research, and had TH DXA scans at baseline and months 6 and 12. aBMD by 2D-DXA was also determined for these women.

The Phase 2 study and its extensions^1^^,^^3^^,^^15^ randomized 419 women aged 55-85 yr with a low BMD (T-score of ≤−2.0 and ≥−3.5 at the LS, TH, or FN) into multiple arms and interventions over a 6-yr period. The primary endpoint was percentage change from baseline in aBMD by DXA at the LS at month 12, and key secondary endpoints included percentage changes from baseline in BMD at the LS at month 6 and at the TH and FN at months 6 and 12.^1^ Results for the primary and secondary endpoints have been previously published.^1^ In the current analysis, we focus on select subpopulations of women who received subcutaneous placebo (52 women), daily subcutaneous teriparatide 20 μg (55 women), or monthly subcutaneous romosozumab 210 mg (52 women) in the first 12 mo of the treatment period of the study^1^ (Figure S1B). 3D-DXA was performed on data from women in these treatment groups who had completed the 12-mo study period, had provided consent for future research, and had TH DXA scans at baseline and months 3, 6, and 12. aBMD by 2D-DXA was also determined for these women.

### 3D-DXA using 3D-SHAPER software

Image files of hip 2D-DXA scans taken at specified time points in STRUCTURE and the Phase 2 study were transferred for the 3D-DXA analysis using the 3D-SHAPER software (v2.11, 3D-SHAPER Medical) as previously described,^7^^,^^8^ with operators blinded to treatment. Briefly, the 3D-SHAPER software uses a statistical model based on a database of 111 QCT scans from Caucasian men (*n* = 30) and women (*n* = 81) to generate a 3D patient-specific model of the proximal femur that allows a separate characterization of the cortical and trabecular bone compartments.^7–9^ Integral vBMD (expressed in mg/cm^3^) was calculated as the mean vBMD of the integral (ie, cortical and trabecular) compartment at the TH region. Cortical bone was segmented by fitting a function of cortical thickness (expressed in mm), cortical vBMD (expressed in mg/cm^3^), location of the cortex, density of surrounding tissues, and imaging blur to the density profile computed along the normal vector at each node of the proximal femur surface mesh.^8^ Cortical surface BMD (sBMD; a measure of density-to-thickness expressed in mg/cm^2^) was calculated as the product of cortical vBMD and cortical thickness at each vertex of the femoral surface of the 3D model.^7^^,^^8^ As cortical sBMD derives from both the cortical thickness and density, it is a surrogate parameter for cortical bone strength.^10^ Mean cortical thickness, mean cortical vBMD, and mean cortical sBMD were computed for the TH region. Trabecular vBMD (expressed in mg/cm^3^) was calculated using the output 3D image as the average vBMD of the trabecular compartment at the TH region.

### Anatomical distribution of changes in bone structure

The 3D data generated from the hip DXA scans in STRUCTURE and the Phase 2 study were used to calculate average 3D models and assess the anatomical distribution of changes in bone structure in each treatment group. One average 3D model per time point (STRUCTURE: baseline, month 6, and month 12; Phase 2 study: baseline, month 3, month 6, and month 12) and treatment group was generated using image registration techniques. Briefly, generalized procrustes analysis (GPA)^16^ was used to align the femoral shapes within the same time point and treatment group. Thin plate spine (TPS) transformations^17^ were calculated between the average shape and the aligned femoral shapes. The transformations from GPA and TPS were applied to the 3D-DXA density images, producing in set of aligned density images, with a shape matching the average femoral shape. For each treatment group, the average 3D models obtained at the specified time points were compared with those at baseline to assess the anatomical distribution of the changes in bone structure and density. Changes in cortical thickness, cortical vBMD, and cortical sBMD were displayed at the periosteal surface of the femur using 3D visualizations; changes in cortical and trabecular vBMD were displayed using cross-sectional images.^12^

### Statistical analysis

For STRUCTURE, treatment group comparisons for percentage change from baseline to months 6 and 12 in aBMD, integral vBMD, cortical thickness, cortical vBMD, cortical sBMD, and trabecular vBMD were assessed by a repeated measures model adjusting for treatment, visit, baseline serum β-isomer of the C-terminal telopeptide of type I collagen (β-CTX) value, baseline value, DXA-machine type, treatment-by-visit interaction, and baseline value-by-machine type interaction. Percentage changes from baseline were reported as least squares (LS) means and associated 95% CIs. No adjustments for multiplicity were made, and there was no imputation of missing data. For the Phase 2 study, percentage changes from baseline to months 3, 6, and 12 in aBMD, integral vBMD, cortical thickness, cortical vBMD, cortical sBMD, and trabecular vBMD were assessed using descriptive summary statistics and reported as means and associated 95% CIs. For STRUCTURE, Pearson correlation (*R*) was used to assess the relationships of data for absolute measurements between TH aBMD by 2D-DXA and integral vBMD by 3D-DXA, and absolute measurements between TH vBMD (integral, cortical, and trabecular) by QCT using QCT-Medical Imaging Analysis Framework software (MIAF-Femur version 6.2.0; University of Erlangen, Germany) as previously published^6^ and by 3D-DXA using 3D-SHAPER software as presented in our current analysis.

## Results

### Patients and baseline characteristics

**Figure 1:**
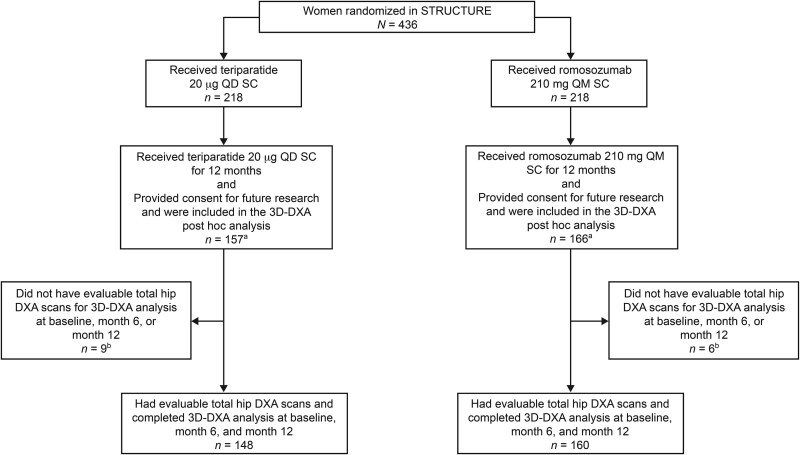
Patient disposition of the subpopulation of STRUCTURE evaluated in the 3D-DXA post hoc analysis. ^a^The STRUCTURE population for the 3D-DXA post hoc analysis included a total of 323 women (teriparatide, 157; romosozumab, 166). ^b^Women were excluded because the DXA scanner or DXA acquisition mode was not supported by the 3D-SHAPER software or because of missing follow-up data points. 3D, three-dimensional; QD, daily; QM, monthly; SC, subcutaneous.

**Figure 2:**
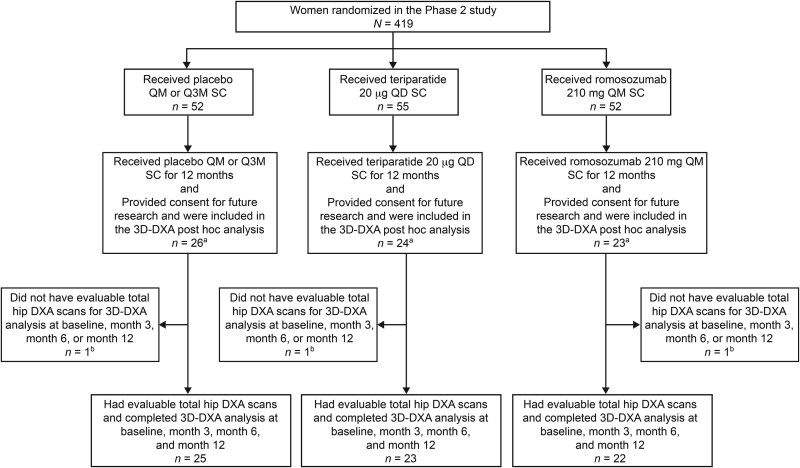
Patient disposition of the subpopulation of the Phase 2 study evaluated in the 3D-DXA post hoc analysis. The Phase 2 dose-ranging study and its extensions randomized 419 women 55-85 yr old with a low BMD (T-score of ≤−2.0 and ≥−3.5 at the LS, TH, or FN) into multiple arms and interventions over a 6-yr period. Data from select subpopulations of women who received placebo QM or Q3M, teriparatide QD, or romosozumab 210 mg QM in the first 12 mo of the treatment period of the study were evaluated (*N* = 159). ^a^Of the 159 women, 73 had provided consent for future research (placebo, 26; teriparatide, 24; romosozumab, 23) and were included in the analysis. ^b^Women were excluded because the DXA scanner or DXA acquisition mode was not supported by the 3D-SHAPER software or because of missing follow-up data points. 3D, three-dimensional; Q3M, every 3 mo; QD, daily; QM, monthly; SC, subcutaneous.

Disposition of patients included in the post hoc analysis for STRUCTURE and the Phase 2 study is illustrated in Figure 1 and Figure 2, respectively.

In STRUCTURE, which enrolled women who had received bisphosphonates for ≥3 yr and alendronate for ≥1 yr before enrollment, 323 of the 436 women had provided consent for future research (3D-DXA: teriparatide, 157; romosozumab, 166) and were included in the analysis (Figure 1). Of the 323 women, 15 were excluded from the analysis (teriparatide, 9; romosozumab, 6) because the DXA scanner or DXA acquisition mode was not supported by the 3D-SHAPER software or because of missing follow-up data points, leaving 308 women who had evaluable 3D-DXA assessments at baseline and months 6 and 12 (teriparatide, 148; romosozumab, 160). Of these 308 women, those with QCT data were included in the QCT analysis (teriparatide, 123; romosozumab, 133).

In the Phase 2 study, there were 159 women in the treatment groups of interest (placebo, 52; teriparatide, 55; romosozumab, 52). Of the 159 women, 73 had provided consent for future research (placebo, 26; teriparatide, 24; romosozumab, 23) and were included in the analysis (Figure 2). Of the 73 women, 3 were excluded because the DXA scanner or DXA acquisition mode was not supported by the 3D-SHAPER software or because of missing follow-up data points (1 patient per each arm), leaving 70 women who had evaluable 3D assessments at baseline and months 3, 6, and 12 (placebo, 25; teriparatide, 23; romosozumab, 22).

Baseline characteristics for the women in the post hoc 3D-DXA-based analysis were consistent with recruitment criteria for each study and consistent with the baseline characteristics for the overall populations for each study (Table 1; Tables S1 and S2). In the STRUCTURE 3D-DXA population, mean (SD) age was 71.4 (7.7) yr and mean (SD) baseline T-scores were −2.9 (1.0) at the LS, −2.2 (0.8) at the TH, and −2.4 (0.7) at the FN (Table 1; Table S1). All women had prevalent vertebral and/or nonvertebral fractures prior to enrollment per the study protocol. Baseline median levels of both procollagen type I N-terminal propeptide (PINP) and β-CTX were low in STRUCTURE, consistent with prior alendronate treatment.^6^ In the Phase 2 study 3D-DXA population, mean (SD) age for the placebo, teriparatide, and romosozumab groups were 66.3 (7.0) yr, 65.8 (6.0) yr, and 64.7 (6.8) yr, respectively (Table 1; Table S2). The corresponding mean (SD) baseline T-scores were −2.3 (0.6), −2.3 (0.5), and −2.4 (0.5) at the LS; −1.2 (0.7), −1.0 (0.9), and −1.3 (0.7) at the TH; and −1.6 (0.6), −1.7 (0.8), and −1.8 (0.6) at the FN. Baseline median levels of both PINP and β-CTX were consistent with those in treatment-naïve patients.^1^

**Table 1:** Baseline characteristics of women included in the STRUCTURE 3D-DXA population and the Phase 2 study 3D-DXA population.

**Characteristic**	**STRUCTURE 3D-DXA population** ^a^		**Phase 2 Study 3D-DXA population** ^b^		
	**Teriparatide** ***n* = 148**	**Romosozumab** ***n* = 160**	**Placebo** ***n* = 25**	**Teriparatide** ***n* = 23**	**Romosozumab** ***n* = 22**
**Age, years, mean ± SD**	71.6 ± 8.1	71.3 ± 7.3	66.3 ± 7.0	65.8 ± 6.0	64.7 ± 6.8
**BMD T-score, mean ± SD**					
** Lumbar spine**	−2.9 ± 1.0	−2.9 ± 1.0	−2.3 ± 0.6	−2.3 ± 0.5	−2.4 ± 0.5
** Total hip**	−2.2 ± 0.8	−2.2 ± 0.8	−1.2 ± 0.7	−1.0 ± 0.9	−1.3 ± 0.7
** Femoral neck**	−2.4 ± 0.7	−2.5 ± 0.7	−1.6 ± 0.6	−1.7 ± 0.8	−1.8 ± 0.6
**Any vertebral fracture, *n* (%)**	148 (100.0)^c^	160 (100.0)^c^	ND	ND	ND
**PINP, μg/L, median (Q1, Q3)**	26.0 (20.0, 33.0)	26.0 (18.0, 34.0)	46.8 (37.6, 55.7)	47.6 (41.7, 63.3)	54.5 (48.1, 68.6)
***β*-CTX, ng/L, median (Q1, Q3)**	231.0 (169.0, 320.0)	217.5 (148.0, 305.5)	421.0 (332.0, 553.0)	432.0 (399.0, 559.0)	517.5 (409.0, 735.0)

*n* = number of women who had completed the 12-mo study period, had provided consent for future research, had evaluable TH DXA scans at baseline and at the time of interest (months 6 and 12 in STRUCTURE; months 3, 6, and 12 in the Phase 2 study), and completed the 3D-DXA analysis.

^a^Patients received oral bisphosphonate therapy for osteoporosis for ≥3 yr, including alendronate for ≥1 yr before starting the study. The mean ± SD duration of prior oral bisphosphonate use was 6.2 ± 2.9 yr while that of prior alendronate use was 5.6 ± 3.2 yr.

^b^Only select treatment groups of the Phase 2 study were included in the current assessment.

^c^All patients in STRUCTURE had historical fracture (ie, nonvertebral fractures after age 50 or vertebral fracture at any time), but fractures were self-reported and not confirmed or adjudicated.

Abbreviations: 3D, three-dimensional; β-CTX, β-isomer of the C-terminal telopeptide of type I collagen; ND, not determined; PINP, procollagen type I N-terminal propeptide; Q, quartiles.

### Mean percentage changes from baseline in hip aBMD (2D-DXA) and integral vBMD (3D-DXA and QCT)

**Figure 3:**
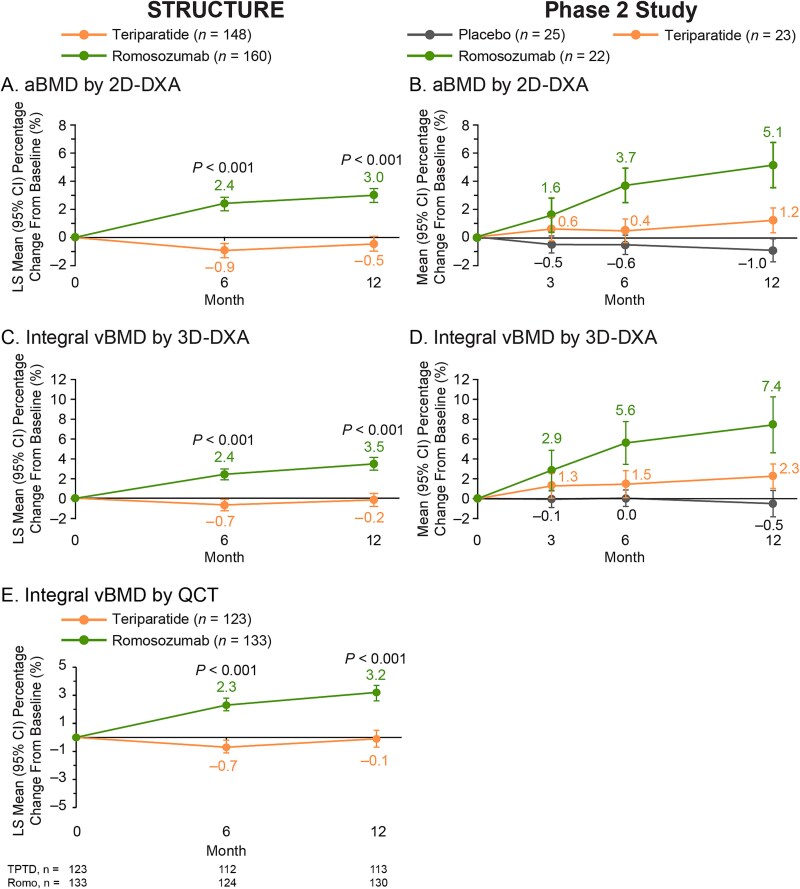
Mean percentage change from baseline to time of interest in aBMD by 2D-DXA, integral vBMD by 3D-DXA, and integral vBMD by QCT in STRUCTURE (A, C, and E) and the Phase 2 study (B and D) from analysis of hip DXA scans. *n* = number of women who had completed the 12-mo study period, had provided consent for future research, had evaluable TH DXA scans at baseline and at the time of interest (months 6 and 12 in STRUCTURE; months 3, 6, and 12 in the Phase 2 study), and completed the 3D-DXA analysis. For 2D-DXA and 3D-DXA, the number of patients with data evaluated at baseline and subsequent months was the same. For QCT, the number of patients with data evaluated at baseline, month 6, and month 12 is shown below the graph. In STRUCTURE, treatment group comparisons for percentage change from baseline for bone parameters were assessed by a repeated measures model adjusting for treatment, visit, baseline serum C-telopeptide of type I collagen value, baseline value, machine type (for 2D-DXA and 3D-DXA only), treatment-by-visit interaction, and baseline value-by-machine type interaction (for 2D-DXA and 3D-DXA only), and using an unstructured variance covariance structure. *p*-values are for the comparison of the two treatment groups in each study. In the Phase 2 study, treatment group comparisons for percentage change from baseline for bone parameters were assessed using descriptive statistics. 2D, two-dimensional; 3D, three-dimensional; aBMD, areal BMD; LS, least squares; Romo, romosozumab; TPTD, teriparatide; vBMD, volumetric BMD.

Integral bone density for STRUCTURE was measured as aBMD by 2D-DXA, and integral vBMD was measured by 3D-DXA and by QCT; integral bone density for the Phase 2 study was measured as aBMD by 2D-DXA, and integral vBMD was measured by 3D-DXA (Figure 3A-E).

In STRUCTURE, treatment with romosozumab vs teriparatide for 6 mo resulted in significantly greater increases in TH aBMD by 2D-DXA (2.4% vs −0.9%; Figure 3A) and similarly resulted in significantly greater increases in estimated integral vBMD by 3D-DXA (2.4% vs −0.7%; Figure 3C) and QCT (2.3% vs −0.7%; Figure 3E) (*p* < .001 for all bone parameters). At month 12, the cumulative gains from baseline were also significantly greater in the romosozumab group than in the teriparatide group for aBMD by 2D-DXA (3.0% vs −0.5%) and for integral vBMD by both 3D-DXA (3.5% vs −0.2%) and QCT (3.2% vs −0.1%) (*p* < .001 for all bone parameters; Figure 3A, C, and E).

In the Phase 2 study, treatment with romosozumab vs teriparatide for 12 mo resulted in numerically greater gains with romosozumab vs teriparatide in both aBMD (5.1% vs 1.2%) and integral vBMD (7.4% vs 2.3%). Differential gains were already observed at 3 mo by 2D-DXA (1.6% vs 0.6%; Figure 3B) and integral vBMD by 3D-DXA (2.9% vs 1.3%; Figure 3D) and at month 6 in both 2D-DXA aBMD and 3D-DXA integral vBMD (3.7% vs 0.4% and 5.6% vs 1.5%, respectively). In contrast, no changes or slight decreases in aBMD and integral vBMD were observed with placebo (Figure 3B and D).

### Mean percentage change from baseline in hip cortical and trabecular bone parameters

**Figure 4:**
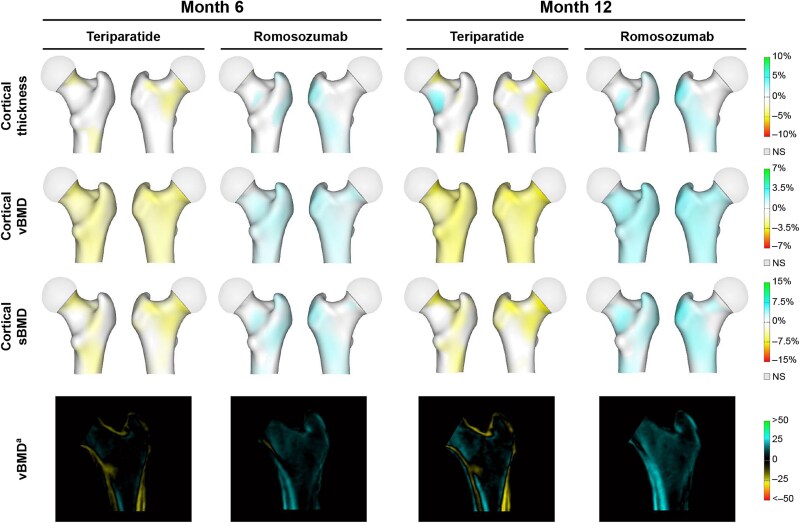
Mean percentage change from baseline to months 6 and 12 in cortical thickness, cortical vBMD, cortical sBMD, and changes in vBMD for cortical and trabecular compartments by 3D-DXA analysis of hip DXA scans in STRUCTURE. Increases in bone parameters are presented in blue-green color; decreases are presented in yellow-red color. Illustrations for each pair of representative femurs per treatment group show the posterior anterior perspective on the left and the anterior posterior perspective on the right. ^a^vBMD images show changes in mg/cm^3^ in the cortical and trabecular compartments; images and data are from a midcoronal slice. 3D, three-dimensional; NS, not significant against baseline, Student’s *t*-test; sBMD, surface BMD; vBMD, volumetric BMD.

**Figure 5:**
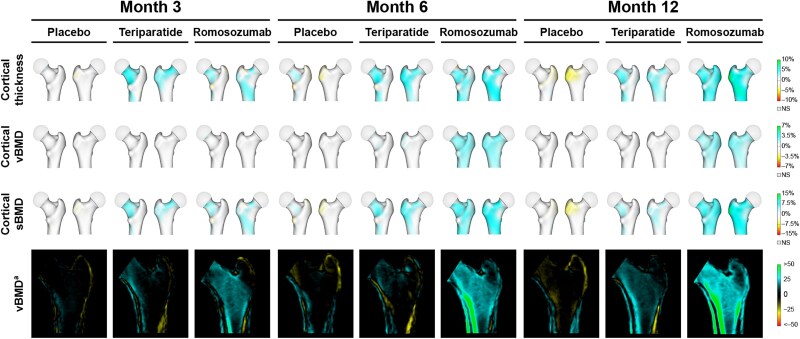
Mean percentage change from baseline to month 3, month 6, and month 12 in cortical thickness, cortical vBMD, cortical sBMD, and changes in vBMD for cortical and trabecular compartments by 3D-DXA analysis of hip DXA scans in the Phase 2 study. Increases in bone parameters are presented in blue-green color; decreases are presented in yellow-red color. Illustrations for each pair of representative femurs per treatment group show the posterior anterior perspective on the left and the anterior posterior perspective on the right. ^a^vBMD images show changes in mg/cm^3^ in the cortical and trabecular compartments; images and data are from a midcoronal slice. 3D, three-dimensional; NS, not significant against baseline, Student’s *t*-test; sBMD, surface BMD; vBMD, volumetric BMD.

**Figure 6:**
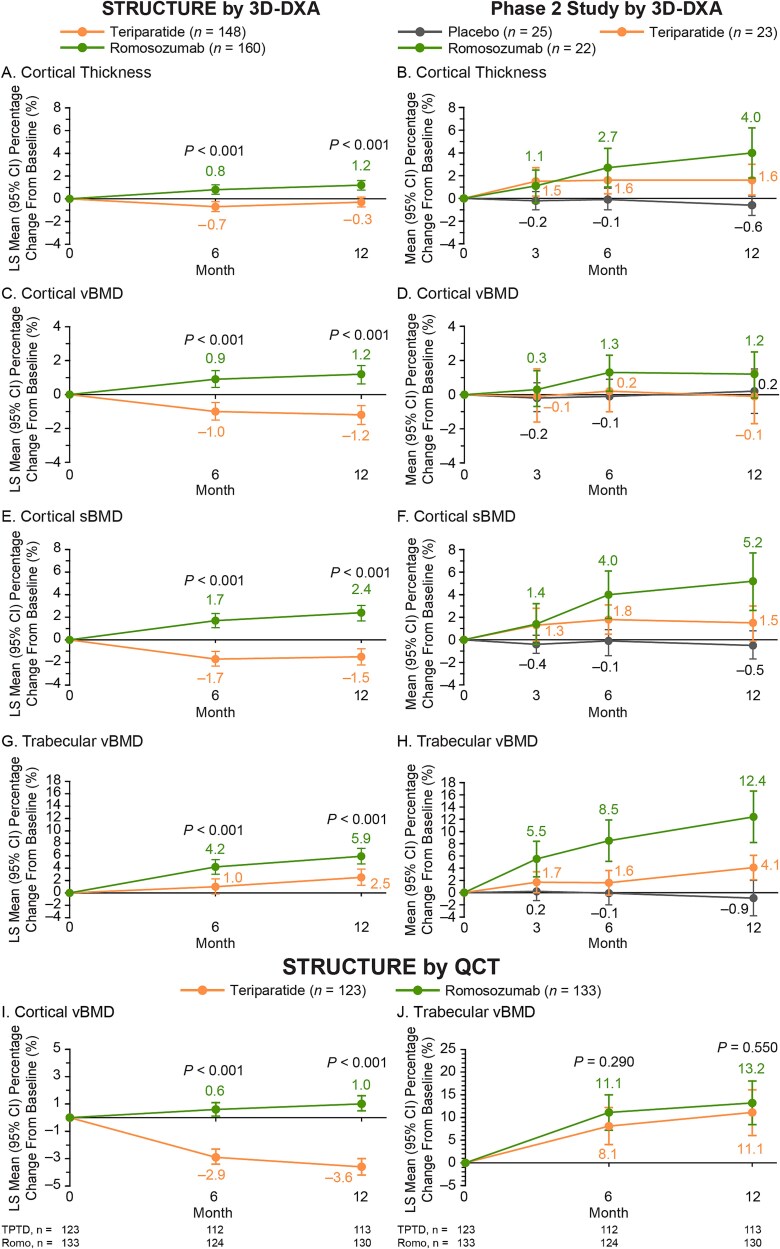
Mean percentage change from baseline to time of interest in cortical thickness, cortical vBMD, cortical sBMD, and trabecular vBMD by 3D-DXA in STRUCTURE (A, C, E, and G) and the Phase 2 study (B, D, F, and H), and cortical vBMD and trabecular vBMD by QCT in STRUCTURE (I and J) from analysis of hip DXA scans. *n* = number of women who had completed the 12-mo study period, had provided consent for future research, had evaluable TH DXA scans at baseline and at the time of interest (months 6 and 12 in STRUCTURE; months 3, 6, and 12 in the Phase 2 study), and completed the 3D-DXA analysis. For 3D-DXA, the number of patients with data evaluated at baseline and subsequent months was the same. For QCT, the number of patients with data evaluated at baseline, month 6, and month 12 is shown below each graph. In STRUCTURE treatment group comparisons for percentage change from baseline for bone parameters were assessed by repeated measures model adjusting for treatment, visit, baseline serum C-telopeptide of type I collagen value, baseline value, machine type (for 3D-DXA only), treatment-by-visit interaction, and baseline value-by-machine type interaction (for 3D-DXA only), and using an unstructured variance covariance structure. *p*-values are for the comparison of the two treatment groups in each study. In the Phase 2 study, treatment group comparisons for percentage change from baseline for bone parameters were assessed using descriptive statistics. 3D, three-dimensional; LS, least squares; Romo, romosozumab; sBMD, surface BMD; TPTD, teriparatide; vBMD, volumetric BMD.

The anatomical distribution of percentage changes from baseline to specified time points in cortical thickness, cortical vBMD, cortical sBMD, and trabecular vBMD by 3D-DXA is shown in Figure 4 for STRUCTURE and Figure 5 for the Phase 2 study, and data for STRUCTURE are illustrated in animated videos in Figure S2 (Video S1). Figure 6 shows graphed LS mean percentage changes in STRUCTURE from baseline to months 6 and 12 in cortical thickness, cortical vBMD, cortical sBMD, and trabecular vBMD by 3D-DXA and cortical vBMD and trabecular vBMD by QCT and mean percentage change in the Phase 2 study from baseline to months 3, 6, and 12 in cortical thickness, cortical vBMD, cortical sBMD, and trabecular vBMD by 3D-DXA.

In STRUCTURE, significant increases were seen by month 6, the first measured time point, following treatment with romosozumab vs decreases observed with teriparatide in estimated cortical thickness (0.8% vs −0.7%), cortical vBMD (0.9% vs −1.0%), and cortical sBMD (1.7% vs −1.7%) by 3D-DXA (*p* < .001 for all parameters; Figure 4; Figure 6A, C, and E; Figure S2 [Video S1]). Additional significant increases were observed through month 12 with romosozumab vs teriparatide in cortical thickness (1.2% vs −0.3%), cortical vBMD (1.2% vs −1.2%), and cortical sBMD (2.4% vs −1.5%) by 3D-DXA (*p* < .001 for all parameters). Consistent with these observations, cortical vBMD by QCT also showed a significant increase with romosozumab while a decrease was confirmed with teriparatide at month 6 (0.6% vs −2.9%) and month 12 (1.0% vs −3.6%) (*p* < .001 for all parameters; Figure 6I). By 3D-DXA, increases in trabecular vBMD were observed with both romosozumab and teriparatide at month 6 (4.2% vs 1.0%) and month 12 (5.9% vs 2.5%), with significantly greater increases observed with romosozumab vs teriparatide at both time points (*p* < .001; Figure 6G). An increase in trabecular vBMD was observed by QCT with both romosozumab and teriparatide at months 6 (11.1% vs 8.1%) and 12 (13.2% vs 11.1%), with no significant difference observed between treatment groups (*p* = .290 at month 6 and *p* = .550 at month 12; Figure 6J).

In the Phase 2 study, at 3 mo, the changes in estimated bone parameters with 3D-DXA were 1.1% and 1.5% for cortical thickness and 1.4% and 1.3% for cortical sBMD with romosozumab and teriparatide, respectively (Figure 6B and F). A numerically greater increase in trabecular vBMD was seen at month 3 following treatment with romosozumab vs teriparatide (5.5% vs 1.7%; Figure 6H). By months 6 and 12, numerically greater increases were observed with romosozumab vs teriparatide for cortical thickness (month 6: 2.7% vs 1.6%, month 12: 4.0% vs 1.6%; Figure 6B), cortical sBMD (month 6: 4.0% vs 1.8%, month 12: 5.2% vs 1.5%; Figure 6F), and trabecular vBMD (month 6: 8.5% vs 1.6%, month 12: 12.4% vs 4.1%; Figure 6H). The percentage change in cortical vBMD with romosozumab vs teriparatide was 0.3% vs −0.1% at month 3, 1.3% vs 0.2% at month 6%, and 1.2% vs −0.1% at month 12 (Figure 6D). Both romosozumab and teriparatide resulted in net increases in cortical thickness, cortical sBMD, and trabecular vBMD, while no changes were observed with placebo overall over 12 mo (Figure 6B, F, and H). For cortical vBMD, no or minimal changes were observed with teriparatide and placebo over 12 mo (Figure 6D).

### Relationship of absolute aBMD (2D-DXA) and absolute integral vBMD (3D-DXA) in STRUCTURE

Since hip 3D-DXA models were based on 2D-DXA hip images, we examined the relationship of absolute aBMD determined by 2D-DXA and absolute integral vBMD determined by 3D-DXA using 3D-SHAPER software in patients in STRUCTURE. There were significant correlations between absolute measurements of integral aBMD obtained by 2D-DXA and integral vBMD by 3D-DXA at baseline, month 6, and month 12, with each *R* > 0.850 for the romosozumab treatment group, the teriparatide treatment group, and both treatment groups combined (Table S3).

### Relationship of data derived by QCT and 3D-DXA in STRUCTURE

To examine the relationship of data obtained by QCT and 3D-DXA, we compared results from patients in STRUCTURE treated with romosozumab or teriparatide who had integral, cortical, and trabecular vBMD data evaluated by both QCT using MIAF software as previously published^6^ and 3D-DXA using 3D-SHAPER software as obtained in the current analysis (Table S4). There were significant correlations between absolute measurements of integral vBMD obtained by QCT and 3D-DXA at baseline, month 6, and month 12, with an *R* of 0.863, 0.841, and 0.867, respectively, in the romosozumab group and *R* of 0.863, 0.864, and 0.866, respectively, in the teriparatide group (*p* < .001 for all treatments and time points; Table S4). *R* for cortical vBMD obtained by QCT and 3D-DXA at baseline, month 6, and month 12 was 0.522, 0.476, and 0.521, respectively, in the romosozumab group and 0.567, 0.616, and 0.550, respectively, in the teriparatide group (*p* < .001 for all treatments and time points; Table S4). *R* for trabecular vBMD obtained by QCT and 3D-DXA at baseline, month 6, and month 12 was 0.838, 0.840, and 0.862, respectively, in the romosozumab group and 0.791, 0.796, and 0.787, respectively, in the teriparatide group (*p* < .001 for all treatments and time points; Table S4).

## Discussion

3D-DXA analysis of standard hip DXA scans using 3D-SHAPER software was applied to assess hip integral, cortical, and trabecular bone changes in patients from the STRUCTURE study and Phase 2 study. Visualization of multiple bone compartments allowed for the evaluation of differential effects of romosozumab and teriparatide treatment through 12 mo, both in the context of treatment-naïve patients (Phase 2 study) and patients who had previously received bisphosphonates and were then treated with romosozumab or teriparatide (STRUCTURE).

Changes in bone parameters were observed as early as 3 mo in treatment-naïve patients and were sustained over 12 mo in both previously treated and treatment-naïve patients. Romosozumab improved trabecular bone (trabecular vBMD) and cortical bone (cortical thickness, cortical vBMD, and cortical sBMD) in previously treated and treatment-naïve patients. Treatment with teriparatide also increased trabecular vBMD in both previously treated and treatment-naïve patients; however, the increases were less than those observed with romosozumab. Notably, teriparatide’s effect on cortical bone was dependent on prior treatment as decreases in cortical bone (cortical thickness, cortical vBMD, and cortical sBMD) were observed with teriparatide in previously treated patients; whereas increases were observed in cortical thickness and cortical sBMD in treatment-naïve patients with teriparatide, albeit to a lower extent compared with romosozumab. Additionally, no change in cortical vBMD was observed with teriparatide in treatment-naïve patients.

Compared with placebo in treatment-naïve patients, romosozumab had greater gains across all parameters evaluated, while teriparatide had greater gains vs placebo in integral vBMD, cortical thickness, cortical sBMD, and trabecular vBMD; relative gains vs placebo were greater with romosozumab vs teriparatide. No changes were observed in cortical vBMD with placebo or teriparatide treatment over 12 mo.

Similar findings with romosozumab were reported in a recent publication evaluating treatment-naïve patients from the ARCH and FRAME studies by 3D-DXA.^9^ In patients from the ARCH study, greater gains in bone parameters were observed with romosozumab vs alendronate treatment for months 12, with the gains sustained after follow-up treatment with open-label alendronate for another 12 mo. In patients from the FRAME study in which patients received romosozumab vs placebo for 12 mo, greater gains in bone parameters were observed with romosozumab and were sustained after follow-up treatment with denosumab for another 12 mo. Taken together, these findings further underscore the clinical importance of treatment sequence, starting with a bone-forming agent and then transitioning to an antiresorptive agent in patients at high risk of fracture.

The different mechanisms of action of romosozumab vs teriparatide may explain the differential effect of the two bone-forming agents on cortical bone. Rooney et al.^18^ demonstrated that the predominantly early effect of teriparatide on bone is due to remodeling-based bone formation (RBBF), although some modeling-based bone formation (MBBF) was observed in the cancellous envelope. Studies in both animal models and humans have reported increases in cortical porosity with teriparatide treatment.^19–22^ Chavassieux et al.^23^ and Eriksen et al.^24^ showed that bone formation with romosozumab at 2 mo was predominantly due to MBBF on endocortical surfaces from iliac crest bone biopsies. Additional studies have shown the net gain in bone mass with romosozumab is due to both MBBF and RBBF in combination with a sustained decrease in surface-based bone resorption leading to continued gains through month 12.^23^^,^^25^ The differential effect of the two bone-forming agents on cortical bone may therefore help explain the observed deterioration of cortical BMD with teriparatide vs romosozumab in patients previously treated with bisphosphates as fewer remodeling sites are available in previously treated bisphosphonate patients for teriparatide to act upon.

A previously published validation study reported high correlations between 3D-DXA and QCT measurements of integral vBMD (*R* = 0.95), cortical vBMD (*R* = 0.93), and trabecular vBMD (*R* = 0.86).^7^ However, in the current study, the strengths of correlations between 3D-DXA and QCT integral vBMD (*R* = 0.84-0.87), cortical vBMD (*R* = 0.48-0.62), and trabecular vBMD (*R* = 0.79-0.86) were found to be lower compared with those reported previously, particularly for the cortical compartment. These lower correlations could be due to QCT-based MIAF software and 3D-SHAPER software using different approaches for cortical segmentation, region of interest definition, and the definition of the trabecular bone. In another study, Dudle et al.^26^ reported high correlations 3D-DXA and QCT measurements of TH integral vBMD (*R^2^* = 0.93 or *R* = 0.96), similar to those reported by Humbert et al.^7^ However, Dudle et al. also found that integral vBMD estimated by 3D-DXA was systematically lower compared to that estimated by QCT. As outlined by the authors, these systematic differences between 3D-DXA and QCT vBMD reported might be explained by: (1) the use of ex vivo scan and water bags to account for soft tissues, which can have a significant impact on DXA measurements and consequently, on 3D-DXA measurements^26^; (2) the use of a solid QCT phantom, which provide density measurement 10%-15% higher, compared with use of liquid phantoms^27^; and (3) the segmentation technique used for QCT images did not correct for partial volume effects, which might explain marked systemic differences in bone volume^28^ compared to the methods used by 3D-SHAPER. The 3D-SHAPER software generates density measurements calibrated using a liquid phantom. Despite these potential differences in 3D-DXA and QCT techniques, the direction of the observations for bone parameter measurements using 3D-DXA in our current study were consistent with results from bone parameter and QCT analyses obtained in the previous STRUCTURE and Phase 2 studies, which included a greater number of patients.^6^^,^^29^ Nevertheless, further investigation is warranted to compare the effect of using solid vs liquid phantoms and ex vivo vs in vivo data in studies comparing 3D-DXA and QCT.

Despite the known limitations of 3D-DXA using 3D-SHAPER software, precision studies have shown that bone parameter measurements using 3D-SHAPER were similar to those of aBMD measurements by DXA.^30^ Similar trend assessment intervals were reported for 3D-SHAPER cortical and trabecular measurement and aBMD measurements, indicating similar performance for monitoring. Additionally, 3D-SHAPER has been used in multiple studies including in patients receiving denosumab, bisphosphonates, teriparatide, abaloparatide, and romosozumab.^9^^,^^11^^,^^12^^,^^31–33^ The results of the studies were found to align with the documented effects of the treatments on integral, cortical, and trabecular bone.

A strength of our analysis is that it used data from two randomized clinical studies with standard assessment of BMD and bone parameters. In STRUCTURE, 308 of 436 women (~71%) were included in the 3D-DXA analysis; thus, these data are representative of the overall STRUCTURE population of patients previously treated with bisphosphonates. In the dose-ranging Phase 2 study, 70 of 159 treatment-naïve women (44%) were included in the 3D-DXA analysis. A limitation of our current study is that we did not analyze whether the changes in cortical or trabecular compartments potentially impacted bone strength to a clinically meaningful extent. The effect of romosozumab on bone strength was previously analyzed using QCT and finite element analysis (FEA) in an analysis of a subset of patients in the Phase 2 study,^34^ and the results demonstrated that romosozumab increased bone strength in the cortical and trabecular components of the LS and hip. Bone strength analysis methods using 3D-DXA and FEA were recently reported and validated against QCT.^26^^,^^35^ It is therefore possible that applying 3D-DXA and FEA to the data in our current study could allow for an investigation into the extent to which changes in the cortical and trabecular compartments influenced bone strength. Another limitation is that although data from the Phase 2 study showed greater gains with romosozumab in bone parameters than with teriparatide or placebo, statistical significance was not determined due to the small number of patients (~20 patients/group). Lastly, 3D-DXA has not been validated using longitudinal data to understand if changes in the bone compartments over time assessed by QCT are similar to changes assessed by 3D-DXA. As a result, caution is required when interpreting longitudinal data derived from 3D-DXA.

3D-DXA has a number of benefits and potential applications in clinical practice. First, DXA scans of patients are readily available in clinical practice, and these can easily be assessed using the 3D-SHAPER software to generate 3D-DXA models for analysis without subjecting patients to additional testing procedures. By comparison, QCT is not widely available in clinical practice, and where available, obtaining QCT scans would require additional testing procedures and exposure of patients to additional radiation. Second, assessment by DXA-based 3D-SHAPER analysis can be performed retrospectively from archived DXA data in studies, where QCT was not obtained. Finally, 3D-DXA can be used to visually monitor the effect of osteoporosis treatments on both the cortical and trabecular bone compartments.

In conclusion, 3D-DXA is a novel and convenient technique that uses standard DXA images to estimate changes in cortical and trabecular bone parameters and can also be used to visualize the anatomical distribution of changes in those parameters to monitor treatment effect. Results from the 3D-DXA analysis of hip DXA scans demonstrated significant improvements in hip cortical and trabecular bone compartments with romosozumab compared with teriparatide in patients previously treated with bisphosphonates from the STRUCTURE study; a similar trend was observed in treatment-naïve patients from the dose-ranging Phase 2 study. These findings support the growing evidence that romosozumab improves hip cortical bone density and structure, thereby contributing to its antifracture efficacy.

## Supplementary Material

Supplementary_Figure_2_Supplementary_Video_1_ziaf151

Supplementary_Material_ziaf151

## Data Availability

Qualified researchers may request data from Amgen clinical studies. Complete details are available at the following: http://www.amgen.com/datasharing.
