# Supplementary material for: Effects of romosozumab and teriparatide on hip bone using 3D-SHAPER in postmenopausal women with osteoporosis
Source: JBMR Plus. 2025 Sep 17;9(11):ziaf151. doi: 10.1093/jbmrpl/ziaf151 (PMC12526953; doi:10.1093/jbmrpl/ziaf151)
Supplement: Supplementary_Material_ziaf151 [file supplementary_material_ziaf151.pdf]

Supplementary Material

Effects of romosozumab and teriparatide on hip bone using 3D-SHAPER in postmenopausal women with osteoporosis

Authors

E Michael Lewiecki,[1] Donald Betah,[2] Ludovic Humbert,[3] Cesar Libanati,[4] Mary Oates,[2] Yifei Shi,[2] Renaud Winzenrieth,[3] Serge Ferrari,[5] and Fumitoshi Omura[6]

Author Institutions

[1]New Mexico Clinical Research & Osteoporosis Center, Albuquerque, NM, USA

[2]Amgen Inc., Thousand Oaks, CA, USA

[3]3D-SHAPER Medical, Barcelona, Spain

[4]UCB, Brussels, Belgium

[5]Geneva University Hospital, Geneva, Switzerland

[6]Koenji Orthopedics Clinic, Tokyo, Japan

Running title

3D modeling from DXA images

Corresponding author

E Michael Lewiecki, MD; New Mexico Clinical Research & Osteoporosis Center, 300 Oak St. NE, Albuquerque, NM 87106, USA; Phone: +1 505-855-5525; E-mail: mlewiecki@gmail.com

ORCID: 0000-0003-2026-9587

Target journal: *JBMR*

Supplementary material: 6 (2 figures and 4 tables)

Supplementary Figure 1. Study designs for STRUCTURE (A) and the Phase 2 dose-ranging study (B).

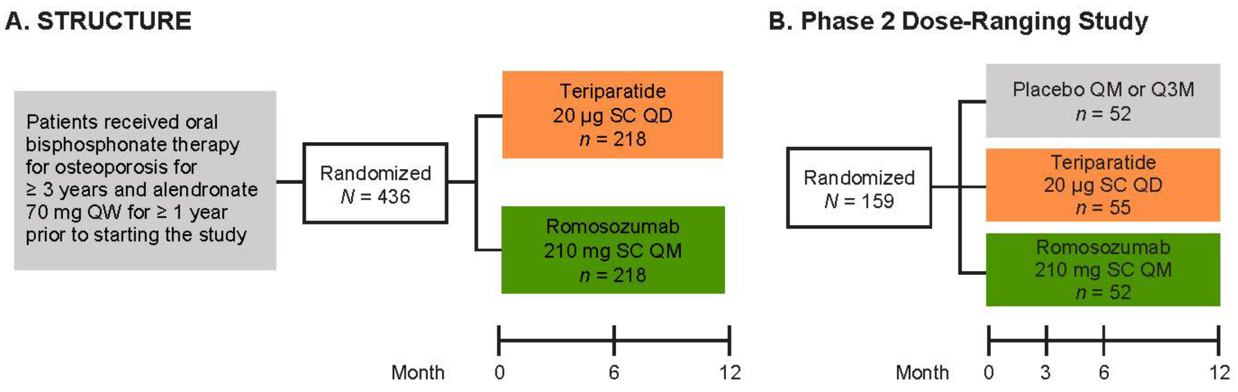

STRUCTURE had enrolled 436 postmenopausal women with osteoporosis who had received oral bisphosphonate therapy for ≥ 3 years and oral alendronate (70 mg weekly or equivalent) for ≥ 1 year prior to screening; women were then randomized 1:1 to receive open-label romosozumab 210 mg SC monthly or teriparatide 20 µg SC daily for 12 months. Data from all enrolled women are included in the current analysis. The mean ± SD duration of prior oral bisphosphonate use was 6.2 ± 2.9 years while that of prior alendronate use was 5.6 ± 3.2 years. The Phase 2 dose ranging study and its extensions randomized 419 women 55–85 years old with a low BMD (T-score of ≤ –2.0 and ≥ –3.5 at the lumbar spine, total hip, or femoral neck) into multiple arms and interventions over a 6-year period. Data from select subpopulations of women who received placebo QM or Q3M, teriparatide 20 µg QD, or romosozumab 120 mg QM in the first 12 months of the treatment period of the study are included in the current analysis (*N* = 159). BMD = bone mineral density; Q3M = every 3 months; QD = daily; QM = monthly; QW = weekly; SC = subcutaneous; SD = standard deviation.

Supplementary Figure 2, Supplementary Video 1. Illustration of computed percentage changes from baseline to months 6 and 12 in cortical thickness, cortical vBMD, cortical sBMD, and vBMD for cortical and trabecular compartments in STRUCTURE

Example: STRUCTURE – Change in Cortical Thickness From Baseline to Month 12

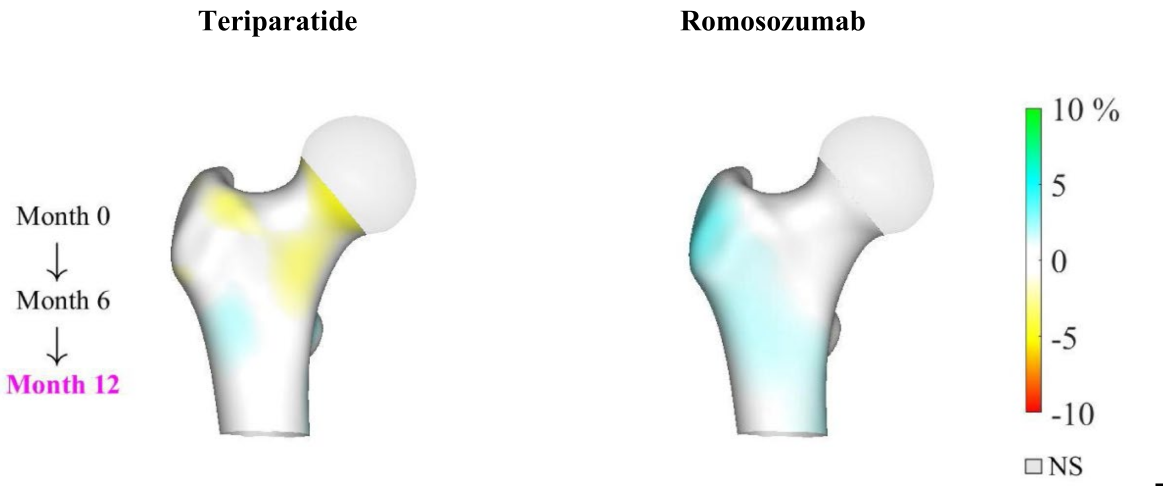

For the images in the videos, increases in bone parameters are presented in the blue-green color; decreases are presented in the yellow-red color. The illustration of the representative femur per treatment group shows and anterior posterior perspective. NS = not significant; sBMD = surface bone mineral density; vBMD = volumetric bone mineral density.

Supplementary Table 1. Baseline characteristics of women included in the STRUCTURE 3D-DXA population and the STRUCTURE overall population

	**STRUCTURE 3D-DXA population**[a,b]		**STRUCTURE overall population**[a]	
	**Teriparatide**	**Romosozumab**	**Teriparatide**	**Romosozumab**
**Characteristic**	***n* = 148**	***n* = 160**	***n* = 218**	***n* = 218**
Age, years, mean ± SD	71.6 ± 8.1	71.3 ± 7.3	71.2 ± 7.7	71.8 ± 7.4
BMD T-score, mean ± SD				
Lumbar spine	–2.9 ± 1.0	–2.9 ± 1.0	–2.9 ± 1.0	–2.8± 1.1
Total hip	–2.2 ± 0.8	–2.2 ± 0.8	–2.2 ± 0.7	–2.3 ± 0.8
Femoral neck	–2.4 ± 0.7	–2.5 ± 0.7	–2.4 ± 0.7	–2.5 ± 0.7
Any historical fracture,[c] n (%)	148 (100.0)	160 (100.0)	217 (99.5)	218 (100.0)
PINP, median (Q1, Q3), μg/L	26.0 (20.0, 33.0)	26.0 (18.0, 34.0)	25.0 (20.0, 33.0)	25.0 (18.0, 34.0)
β-CTX, median (Q1, Q3), ng/L	231.0 (169.0, 320.0)	217.5 (148.0, 305.5)	235.0 (170.0, 320.0)	228.0 (152.0, 313.0)

[a]Patients received oral bisphosphonate therapy for osteoporosis for 3 years, including alendronate for the year before starting the study. The mean ± SD duration of prior oral bisphosphonate use was 6.2 ± 2.9 years while that of prior alendronate use was 5.6 ± 3.2 years. [b]Women who had completed the 12-month study period; had provided consent for future research; had evaluable total hip DXA scans at baseline and at the time months 6 and 12; and completed 3D-DXA analysis. [c]All patients in STRUCTURE had historical fracture (ie, nonvertebral fractures after age 50 or vertebral fracture), but fractures were self-reported and not confirmed or adjudicated; 1 patient in the STRUCTURE overall population did not

have historical fracture. 3D = three-dimensional; β-CTX = β-isomer of the C-terminal telopeptide of type I collagen; BMD = bone mineral density; DXA = dual-energy x-ray absorptiometry; PINP = procollagen type I N-terminal propeptide; Q = quartile; SD = standard deviation.

Supplementary Table 2. Baseline characteristics of women included in the Phase 2 3D-DXA population and select treatment groups of the Phase 2 population

	Phase 2 Study: 3D-DXA population[a,b]			Phase 2 study population[a]		
**Characteristic**	**Placebo** *n* = 25	**Teriparatide** *n* = 23	**Romosozumab** *n* = 22	**Placebo** *n* = 52	**Teriparatide** *n* = 55	**Romosozumab** *n* = 52
Age, years, mean ± SD	66.3 ± 7.0	65.8 ± 6.0	64.7 ± 6.8	67.0 ± 6.5	66.8 ± 5.7	66.3 ± 6.5
BMD T-score, mean ± SD						
Lumbar spine	–2.3 ± 0.6	–2.3 ± 0.5	–2.4 ± 0.5	–2.3 ± 0.7	–2.3 ± 0.6	–2.3 ± 0.6
Total hip	–1.2 ± 0.7	–1.0 ± 0.9	–1.3 ± 0.7	–1.4 ± 0.7	–1.3 ± 0.8	–1.5 ± 0.7
Femoral neck	–1.6 ± 0.6	–1.7 ± 0.8	–1.8 ± 0.6	–1.8 ± 0.6	–1.8 ± 0.7	–1.9 ± 0.6
PINP, µg/L, median (Q1, Q3)	46.8 (37.6, 55.7)	47.6 (41.7, 63.3)	54.5 (48.1, 68.6)	48.0 (38.0, 59.0)	49.0 (42.0, 67.0)	53.0 (42.0, 64.0)
β-CTX, ng/L, median (Q1, Q3)	421.0 (332.0, 553.0)	432.0 (399.0, 559.0)	517.5 (409.0, 735.0)	481.0 (347.0, 673.0)	506.0 (410.0, 690.0)	519.0 (405.0, 642.0)

n = number of women in each treatment group. [a]Only select treatment groups of the Phase 2 study were included in the assessment. [b]Women who had completed the 12-month study period; had provided consent for future research; had evaluable total hip DXA scans at baseline and at months 3, 6, and 12; and completed the 3D-DXA analysis. 3D = three-dimensional; β-CTX = β-isomer of the C-terminal telopeptide of type I collagen; BMD = bone mineral density; DXA = dual-energy x-ray absorptiometry; PINP = procollagen type I N-terminal propeptide; Q = quartile; SD = standard deviation.

Supplementary Table 3. Relationship of total hip absolute aBMD by 2D-DXA and absolute integral vBMD by 3D-DXA

	**Absolute aBMD and absolute integral vBMD**[a,b]					
	**Teriparatide** **n = 148**		**Romosozumab** **n = 160**		**All** **n = 308**	
**Visit**	**Pearson correlation coefficient (*R*)**	**Pearson correlation coefficient *P*-value**	**Pearson correlation coefficient (*R*)**	**Pearson correlation coefficient *P*-value**	**Pearson correlation coefficient (*R*)**	**Pearson correlation coefficient *P*-value**
Month 0	0.900	< 0.001	0.887	< 0.001	0.892	< 0.001
Month 6	0.901	< 0.001	0.887	< 0.001	0.893	< 0.001
Month 12	0.904	< 0.001	0.886	< 0.001	0.894	< 0.001

n = number of women in each treatment group. [a]Absolute aBMD values are in mg/cm^2. [b]Absolute vBMD values are in mg/cm^3. 2D = two dimensional; 3D = three dimensional; aBMD = areal bone mineral density; DXA = dual-energy X-ray absorptiometry; *R* = correlation coefficient; vBMD = volumetric bone mineral density.

Supplementary Table 4. Relationship of total hip absolute integral, cortical, and trabecular vBMD by QCT and by 3D-DXA

Visit	Treatment	Absolute vBMD By QCT,[a] mean (SD) [*n*]	Absolute vBMD By 3D-DXA,[a] mean (SD) [*n*]	Pearson correlation coefficient (*R*) [*n*]	Pearson correlation coefficient *P*-value
Integral					
Month 0	Teriparatide	194.5 (34.4) [174]	240.6 (38.6) [148]	0.863 [123]	< 0.001
	Romosozumab	194.9 (38.9) [173]	237.7 (40.4) [160]	0.863 [133]	< 0.001
Month 6	Teriparatide	193.3 (34.4) [156]	238.8 (37.7) [148]	0.864 [112]	< 0.001
	Romosozumab	198.6 (39.2) [163]	243.0 (41.4) [160]	0.841 [124]	< 0.001
Month 12	Teriparatide	194.1 (33.4) [159]	240.0 (38.1) [148]	0.866 [113]	< 0.001
	Romosozumab	201.9 (40.3) [163]	245.6 (42.1) [160]	0.867 [130]	< 0.001
Cortical					
Month 0	Teriparatide	475.8 (57.5) [174]	729.1 (63.8) [148]	0.567 [123]	< 0.001
	Romosozumab	472.8 (64.3) [173]	723.8 (61.0) [160]	0.522 [133]	< 0.001
Month 6	Teriparatide	466.0 (57.1) [156]	721.6 (65.6) [148]	0.616 [112]	< 0.001
	Romosozumab	476.3 (63.0) [163]	729.4 (59.9) [160]	0.476 [124]	< 0.001
Month 12	Teriparatide	458.8 (56.0) [159]	719.9 (67.5) [148]	0.550 [113]	< 0.001
	Romosozumab	478.4 (64.1) [163]	731.3 (59.8) [160]	0.521 [130]	< 0.001

Visit	Treatment	**Absolute vBMD**<br>**By QCT,[a] mean (SD) [*n*]**	**Absolute vBMD**<br>**By 3D-DXA,[a] mean (SD) [*n*]**	**Pearson correlation coefficient (*R*) [*n*]**	**Pearson correlation coefficient *P*-value**
Trabecular					
Month 0	Teriparatide	49.6 (24.4) [174]	119.5 (28.3) [148]	0.791 [123]	< 0.001
	Romosozumab	50.5 (28.2) [173]	120.0 (29.5) [160]	0.838 [133]	< 0.001
Month 6	Teriparatide	50.4 (23.2) [156]	120.7 (27.6) [148]	0.796 [112]	< 0.001
	Romosozumab	52.5 (27.6) [163]	124.8 (30.6) [160]	0.840 [124]	< 0.001
Month 12	Teriparatide	53.3 (24.7) [159]	122.5 (28.2) [148]	0.787 [113]	< 0.001
	Romosozumab	54.6 (29.5) [163]	126.7 (30.8) [160]	0.862 [130]	< 0.001

n = number of women in each treatment group. [a]Absolute vBMD values are in mg/cm^3. 3D = three-dimensional; CI = confidence interval; DXA = dual-energy x-ray absorptiometry; QCT = quantitative computed tomography; SD = standard deviation; vBMD = volumetric bone mineral density.
